# Bone Mesenchymal Stem Cells Contributed to the Neointimal Formation after Arterial Injury

**DOI:** 10.1371/journal.pone.0082743

**Published:** 2013-12-09

**Authors:** Mincai Li, Suqin Li, Liangzhu Yu, Jiliang Wu, Tonghui She, Yaping Gan, Zhenwu Hu, Wenli Liao, Hongli Xia

**Affiliations:** 1 Hubei Province Key Laboratory on Cardiovascular, Cerebrovascular, and Metabolic Disorders, HuBei University of Science and Technology, Xianning, China; 2 The Medical School, HuBei University of Science and Technology, Xianning, China; 3 Department of Abdominal Surgery, the Central Hospital of Xianning, Xianning, China; University of Torino, Italy

## Abstract

**Objectives:**

Recent findings suggest that in response to repair-to-injury bone marrow mesenchymal stem cells (BMSCs) participate in the process of angiogenesis. It is unclear what role BMSCs play in the structure of the vessel wall. In present study, we aimed to determine whether BMSCs had the capacity of endothelial cells (ECs).

**Methods:**

BMSCs were separated and cultured. FACS and RT-PCR analysis confirmed the gene expression phenotype. The capacity of migration and adhesion and the ultrastructure of BMSCs were examined. The effect of BMSCs transplantation on the vascular repair was investigated in a murine carotid artery-injured model.

**Results:**

BMSCs could express some markers and form the tube-like structure. The migration and adhesion capacity of BMSCs increased significantly after stimulated. In addition, BMSCs had the intact cell junction. *In* *vivo* the local transfer of BMSCs differentiated into neo-endothelial cells in the injury model for carotid artery and contributed to the vascular remodeling.

**Conclusion:**

These results showed that BMSCs could contribute to neointimal formation for vascular lesion and might be associated with the differentiation into ECs, which indicated the important therapeutic implications for vascular diseases.

## Introduction

Under physiological stress or pathological stimuli such as atherosclerosis (AS), vascular proliferation and remodeling become vital events [1]. Recently, some research evidences suggest that stem cells or endothelial progenitor cells (EPC) are involved in the vascular remodeling and repair after arterial injury and angiogenesis. It therefore seems that the injured tissues might specifically attract stem cells such as bone mesenchymal stem cells (BMSCs) and mediate their migration and recruitment [2]. These injured tissues such as thrombus, ischemic zones and sites of arterial injury have been committed by the increasing role of stem/progenitor cells toward [3]. It has been hypothesized that these stem/progenitor cells may be involved in the vascular remodeling after artery injury and be associated with angiogenesis [4,5]. 

The potential application of stem-cell-based therapies for the treatment of cardiovascular diseases is still in a preliminary stage. Some have reported that the transplantation of progenitor cells derived from bone marrow or circulating blood significantly increased left ventricular ejection fraction after myocardial infarction and improved myocardial survival [6-8] . Some have demonstrated that transfer of autologous bone-marrow-cells enhanced improvement of left ventricular systolic function in patients after acute myocardial infarction [9,10] . These findings suggest that BMSCs have a therapeutic potential in the angiogenesis for some coronary or peripheral artery disease. However, recent data indicate that the potential success of stem-cell-based therapy may depend on the functional properties of transplanted cells, and there are some potential challenges, such as the cell isolation techniques for transplantation, scale capacity, reproducibility, and clinical application possibilities [11]. Further understanding of these mechanisms may contribute to the effect for stem-cell-based therapeutic angiogenesis.

Given the key role of stem/progenitor cells in vascular remodeling and angiogenesis after arterial injury, we examined the effect of BMSCs on angiogenesis in vitro and in vivo. The purpose of this study is: i) identification of BMSCs’ markers; ii) function and ultrastructural examination in BMSCs; and iii) assessment of the angiogenesis by providing BMSCs transplantation for injured carotid arteries.

## Experimental Procedures

### Materials

Anti- PECAM-1, anti-vWF and anti-KDR antibody were purchased from Santa Cruz Biotechnology (Santa Cruz, CA, USA); Anti-CD34, CD45, CD107, Sca-1 antibody were from Abcam (Cambridge, UK). All secondary antibodies were from Proteintech Group (USA). Matrigel were purchased from Sigma (St Louis, MO, USA). All animals’ procedures were conducted in accordance with the National Institutes of Health Guide for the Care and Use of Laboratory Animals (NIH publication 85-23, revised 1996) and approved by the institutional animal care and use committee at the Hubei University of Science and Technology, China.

### Isolation and Culture of BMSCs

BMSCs were separated as previously described[12]. Bone marrow cells were isolated from tibias and femurs of 2-month female C57/BL6 and were suspended in Dulbecco's modified Eagle medium (DMEM) with low glucose supplemented with 10% FBS, 1% penicillin-streptomycin, and L-glutamine (2 mM). After cultured for 24 h, non-adherent cells were removed by medium change and fresh medium added to the cells. Medium was changed every 3 days. At passage 3, the cells were harvested and washed with PBS and were characterized by immunofluorescence staining for the stem cell marker such as CD44, c-kit, CD105 and Sca-1. After passage 6, the cells were harvested for the following experiments. 

### FACS Analysis of BMSCs

FACS analysis was performed as previously described[13]. Briefly, BMSCs were cultured to confluence at passage 3 and were collected with trypsin-EDTA and incubated with the primary antibody followed by PE-conjugated with the corresponding secondary antibody. Isotype-identical antibodies served as controls to exclude nonspecific binding. Quantitative analysis was performed using the FACStar flow cytometry and CellQuest software (Becton Dickinson).

### RT-PCR

We performed RT-PCR as previously described[14]. After passage 6, BMSCs were treated with or without 10ng/ml VEGF for 24 h, total cellular RNA was extracted. RNA was reverse-transcribed into cDNA. RT-PCR primers were designed by Beacon Designer 2.1 software. The primer sequence used for ICAM-1-1 was AGGTATCCATCCATCCCAGAGA (forward) and GAGCTCATCTTTCAGCCACTGA (reverse). The primer sequence used for VCAM-1 was ACGGTAAAGAGGTCACTGGG (forward) and GACCCCAATGAAGAAACAGG (reverse). mRNA levels were normalized by using GAPDH as a housekeeping gene (forward: CCAATCAGCTTGGGCTAGAG; reverse: CCTGGGAAAGGTGTCCTGTA) and compared with levels in the mouse universal gene. This relative value of target genes to endogenous reference is described as the fold of GAPDH=2^–ΔCt^.

### Transwell Migration Assay

BMSCs were analyzed for migration using a transwell system (Corning Costar, USA) as described previously [15]. Briefly, 1×10^5^ BMSCs in 100 μL free-serum media (DMEM+1% FBS) were added to the top chamber of 8 μm Costar polycarbonate transwells, while in the bottom chamber, 600 μL 100 nmol/L recombinant human SDF-1α was or wasn’t added. In the positive group, mouse aorta endothelium cells (MAECs) were added to the top chamber. After migration for 1 h (37°C, 100% humidity, 5% CO2 in air), cells on the upper membrane surface were removed and migratory cells on the membrane underside were fixed with 5% (wt/vol) glutaraldehyde and stained using 0.1% (wt/vol) crystal violet solution. Filter inserts were inverted and numbers of migratory cells on the membrane underside were determined, at room temperature, by visualizing the crystal violet-stained cells directly on insert undersides by microscopy. Data were presented as the average number of migratory cells in 5 high-power fields (200×). Each experiment was performed in triplicate, and then the data were averaged for statistical analysis.

### Adhesion Assay Under Static Conditions

The 96-well plates was preincubated with 20 μg/mL fibronectin (FN) fragment (Takara Shuzo Co., Japan) for 4 h. BMSCs treated with TNF-α or without TNF-α (10 U/mL for 6 hours) were added at a density of 5 × 105 cells/well for 45 min at 37°C. In the positive group, MAECs were added. Afterwards, non-adhesive BMSCs were removed by gently washing with prewarmed RPMI 1640 medium for 3 times. Numbers of BMSCs adherent to fibronectin were calculated in 8 microscopic 200-time fields and averaged.

### Transmission Electron Microscopy

Ultrastructural studies were performed as previous described[16]. For transmission electron microscopic observation, the BMSCs on the filters were fixed with 2.5% glutaraldehyde for 4 h, followed by 1% OsO_4_ for 1 h. The specimens were dehydrated with a graded ethanol series and isoamyl acetate. They were desiccated by the critical-point method using CO_2_ and were coated with metal in an IB-3-type ion coater (EIKO Engineering Co Ltd.). Ultrathin sections were cut with an ultramicrotome (MT-7000, Research and Manufacturing) and examined with a FEI Tecnai G^2^ scanning electron microscope at an accelerating potential of 80 kV after being counterstained with uranyl acetate and lead citrate.

### Mouse Artery-injured and Cell Delivery

In this experiment, we labeled BMSCs with bromodeoxyuridine (BrdU) at a 1mg/ml dilution for 40 min at room temperature to trace these cells in vivo as described previously[17]. All animal experiments were performed according to protocols approved by the Institutional Committee for Use and Care of Laboratory Animal. Briefly, twelve 6-week C57BL/6J female were anesthetized with 50 mg/kg intraperitoneal pentobarbital and a unilateral carotid artery was injured by removal of endothelium with a flexible wire as previously described[18]. 48 h after surgery, 6 mice were intravenous injected 1x10^5^ BMSCs in 100 µL medium , while control groups were administered with 100 µL medium without BMSCs .

### Morphometric Analysis of the Transplanted-BMSC Detection

Four days after carotid artery injured, mice were sacrificed and the arteries were harvested, fixed by formalin and used for detection of BrdU and PECAM-1 by immunofluorescence staining. For detection of BrdU and PECAM-1, a marker endothelial cell, the immunofluorescence staining was used. The procedure was similar to that described previously[19]. Mainly, the specimens were incubated with the anti-BrdU or anti-PECAM-1 antibody for 90 minutes then incubated with secondary antibody for 1 h. 

### Statistical Analysis

All results were presented as mean ± SEM of at least three independent experiments. Statistical significance was evaluated using the unpaired Student’t test for comparisons between two means and was analyzed by ANOVA and post hoc Tukey's test for multiple comparisons between groups . A probability value of P<0.05 was considered significant. 

## Results

### Characteristics of BMSCs

**Figure 1:**
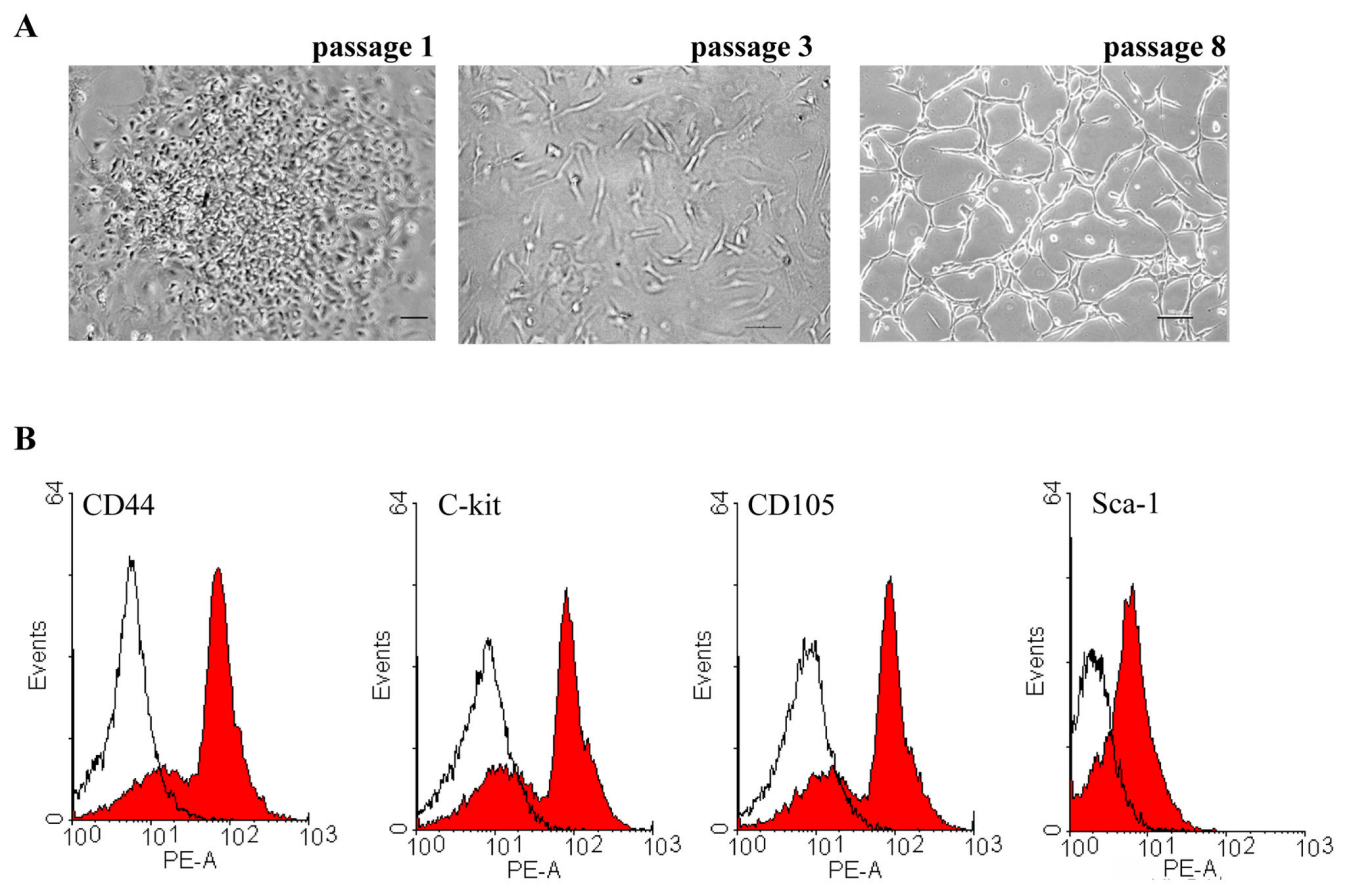
Characteristics of BMSCs. (A) Representative images showed the morphology of BMSCs at passage 1, 3 and 8 under a microscope. Scale bars represent 100μm. (B) Representative images of FACS analysis showed that BMSCs expressed some maker for CD44, c-kit, CD105 and Sca-1. Isotype control staining was shown in shade in representative histograms (n = 3).

Morphologically, BMSCs displayed clusters in an undifferentiated status at first (passage 1). After passage 3, BMSCs displayed a monolayer in medium condition. After passage 8, BMSCs grew with a round, long fiscal, spindle-shaped or whirlpool-shaped appearance, which was densely interconnected (Figure 1A). We investigated whether the BMSCs expressed some stem cell markers at passage 3. These BMSCs were detected for CD44, c-kit, CD105 and Sca-1 by the FACS analysis. As shown in Figure 1C, the expressions of marker gene for stem cell were positive. 

### The Tube-like Formation and the Junction of BMSCs

**Figure 2:**
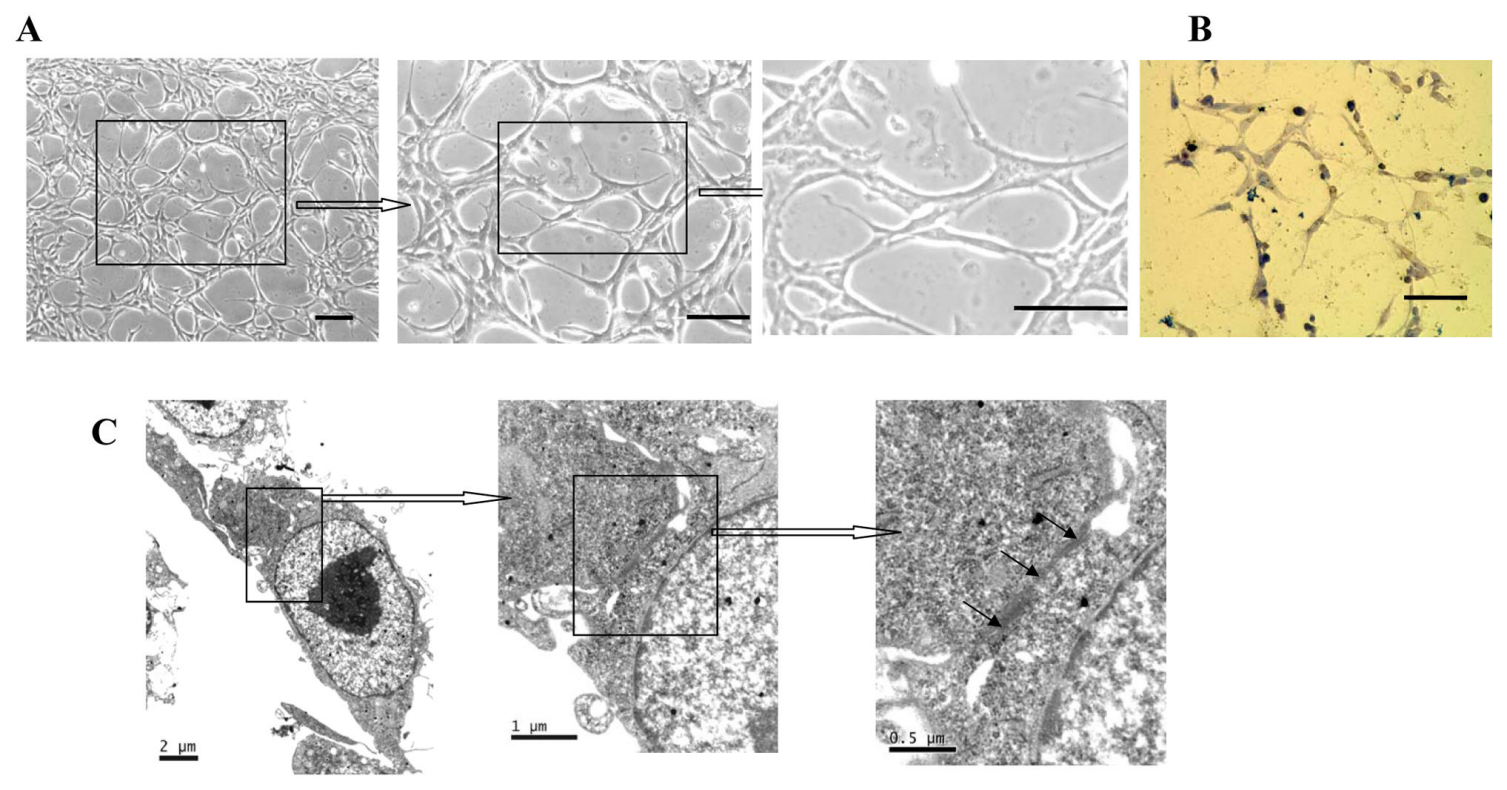
The tube formation and the ultrastructure of BMSCs. (A) The respective photomicrographs showed the tube formation among BMSCs. Scale bars represent 100μm. (B) BMSCs form the capillary tube-like structure on Matrigel. The respective photomicrograph is tube formation on Matrigel for 8 h. Scale bars represent 100μm. (C) The ultrastructure of BMSCs under transmission electron microscopy. The respective photomicrographs showed the junctions among the membrane of BMSCs and the electron-dense of the cell junctions (arrowheads).

After passage 10, BMSCs displayed and formed the tube-like structure and the formation of capillaries tube-like structure, which was found and unaltered in size among BMSCs (Figure 2A). These morphology showed that BMSCs had the capacity of the tube formation after cultured. As shown in Figure 2B, BMSCs could form capillaries tube-like structure on Matrigel.

We confirmed an intact junction structure in BMSCs by transmission electron microscopy analysis. As shown in Figure 2C, BMSCs displayed junction of interconnected cells between BMSCs. The junctions include tight junctions and adherens junctions. Tight junctions comprise three types of transmembrane proteins, occludins, claudins and junctional adhesion molecule. Adherens junctions are ubiquitous along the vascular tree and are formed by transmembrane proteins belonging to the cadherin superfamily. ECs have adherens junctions and tight junctions similar to the epithelial cells. BMSCs showed the cell–cell junction like ECs. Endothelial cell-to-cell junctions are complex structures formed by different adhesive molecules [20,21]. 

### BMSCs Express Some Marker of Endothelial Cell

**Figure 3:**
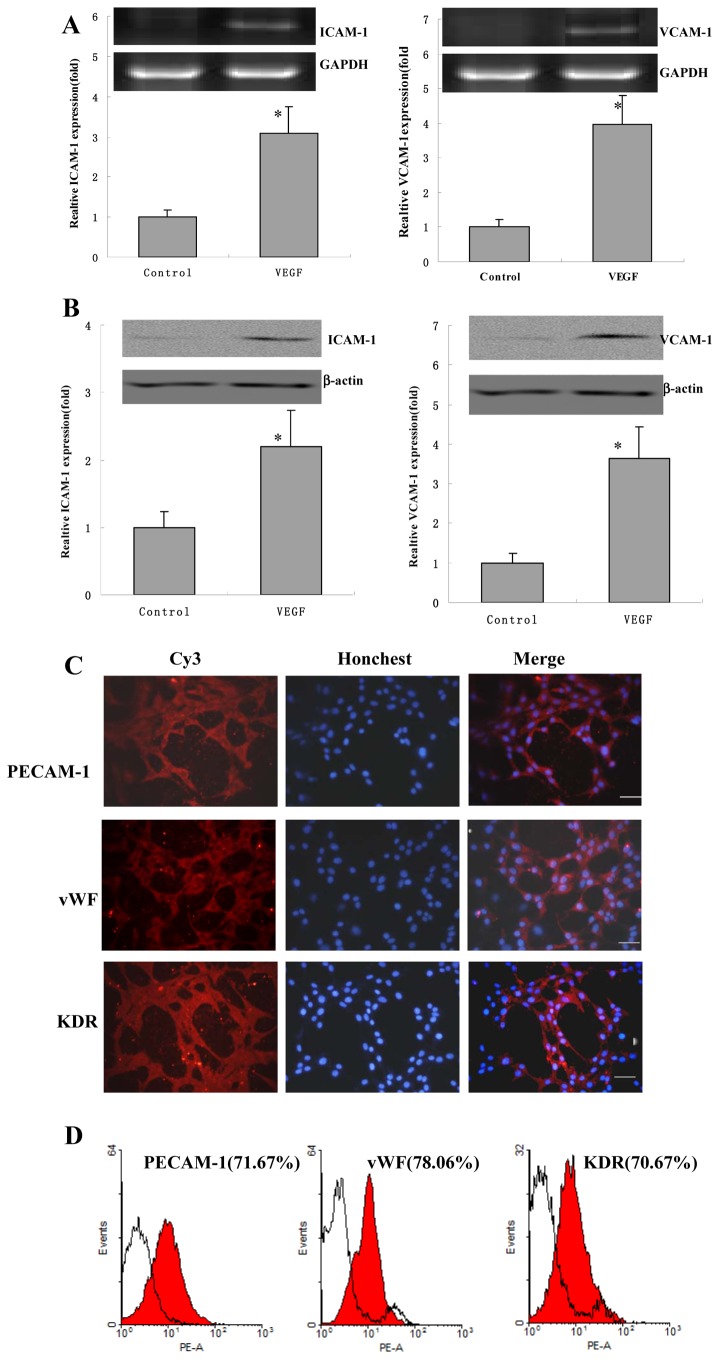
The mRNA expression levels of ICAM-1 and VCAM-1 in BMSCs. (A) BMSCs were treated with or without 10 ng/ml VEGF for 24 h. Relative ICAM-1 and VCAM-1 mRNA expression levels were determined by RT-PCR. Values of mRNA amounts were normalized to GAPDH expression and expressed relative to control (n=6). Error bars represent SEM (* P<0.05). (B) Relative ICAM-1 and VCAM-1 protein expression levels were determined by western blotting. Values of protein amounts were normalized to β-actin expression and expressed relative to control (n=3). Error bars represent SEM (* P<0.05). (C) The immunofluorescence analysis of PECAM-1, vWF and KDR expression in BMSCs. BMSCs were stained for KDR, vWF and PECAM-1 with Cy3 and were stained for nucleus with Honchest33258. Scale bars represent 100μm. (D) The FACS analysis of PECAM-1, vWF and KDR expression in BMSCs and the quantification were counted. Isotype control staining was shown in shade in representative histograms (n = 3).

After passage 6, BMSCs were treated with or without 10 ng/ml VEGF for 24 h, and RT-PCR was used to quantify mRNA levels of intercellular adhesion molecule (ICAM) -1, is an immunoglobulin (Ig)-like cell adhesion molecule expressed by several cell types including endothelial cells and leukocytes[22]. Compared with controls, ICAM-1 mRNA expression was significantly upregulated 3.0±0.6 fold. The expression mRNA level of VCAM-1, an adhesion molecule expressed on endothelial cells, was examined in BMSCs. The levels of VCAM-1 were significantly upregulated 3.9±0.8 fold compared with control groups (Figure 3A). As compared with controls, ICAM-1 protein expression was significantly increased 2.2±0.5 fold. The levels of VCAM-1 were significantly increased 3.7±0.8 fold compared with control groups (Figure 3B).

**Figure 4:**
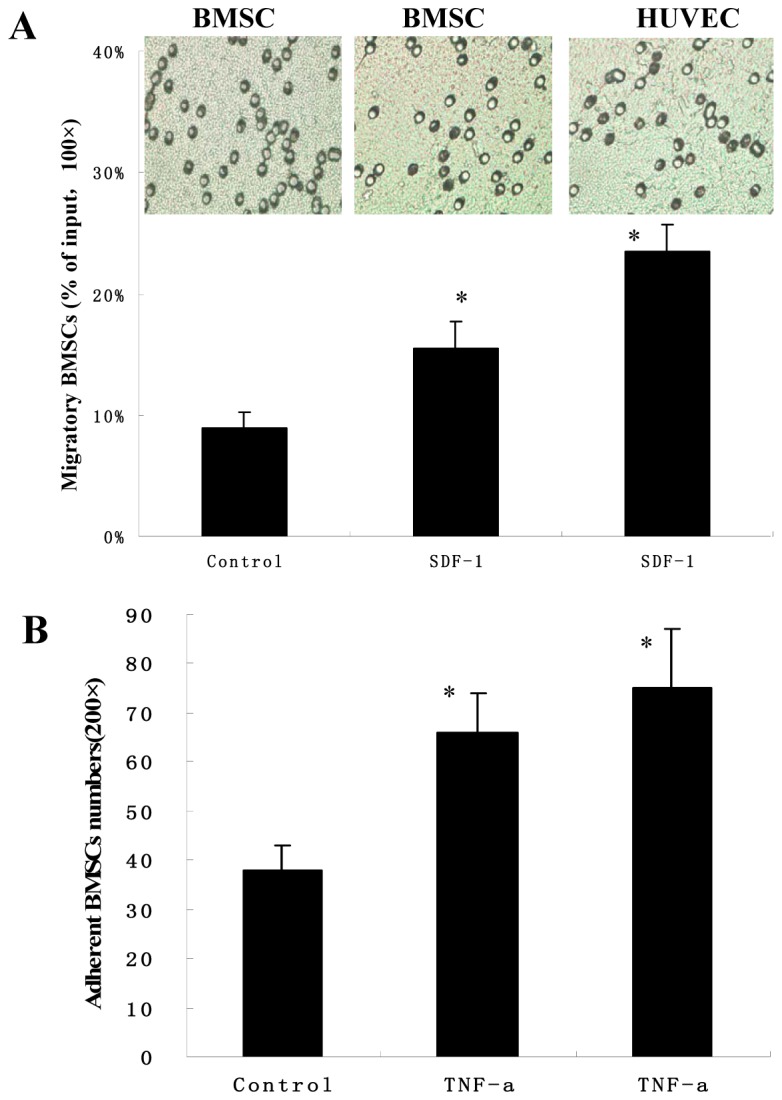
The migration and the adhesion capacity of BMSCs. (A) The migration capacity was examined by the transwell migration assays. The BMSCs were attracted with or without SDF-1 on the inserts and were stained with crystal violet (upper). Quantitative data of the migratory BMSCs were counted in 5 high-power fields (lower). MAEC was the positive group. Values are means±SEM. *p<0.05 versus control group. Results are representative of three separate experiments. (B) The adhesion capacity was examined by TNF-α on fibronectin. The adhesion BMSCs on the fibronectin were counted in 8 different fields. MAEC was the positive group. Values are means±SEM. *p<0.05 versus control group. Results are representative of three separate experiments.

We investigated whether BMSCs express markers such as PECAM-1, vWF and KDR. The PECAM-1 is a glycoprotein localized to the endothelial cell junction where it forms Ca^2+^-independent cell-cell adhesions and it is used as a mature endothelial cell linage-specific marker [23,24]. The vWF is a glycoprotein of protomeric subunits derived from endothelial cells that promoted platelet adhesion[25]. As shown in Figure 3C, BMSCs were positive for PECAM-1, vWF and KDR (the EC linage-specific marker) in immunofluorescence analysis. The quantification positive of PECAM-1, vWF and KDR by FACS analysis were shown in Figure 4D. These results suggested that BMSCs expressed some markers for endothelial cell.

### The Capacities of Migration and Adhesion of BMSCs

To determine whether BMSCs had the ability of the migration of endothelial cell, transwell migratory assay was examined (Figure 4A). The assay showed that the BMSCs had the migratory response to the chemokines SDF-1. The migration of BMSCs in the SDF-1 group vs control group was 16.2%±3.4% vs 9.9%±1.3%, respectively. SDF-1 significantly enhanced the migration of BMSCs like MAECs. We investigated adhesion ability of BMSCs to fibronectin. As shown in Figure 4B, Compared with the control group, BMSCs treated with TNF-α had the significant effect on the adhesion to fibronectin and the adherent number of BMSCs showed a markedly improvement. These data suggested that BMSCs had the capacity of the migration and adhesion.

### Local Transfer of BMSCs Increased neointimal formation

**Figure 5:**
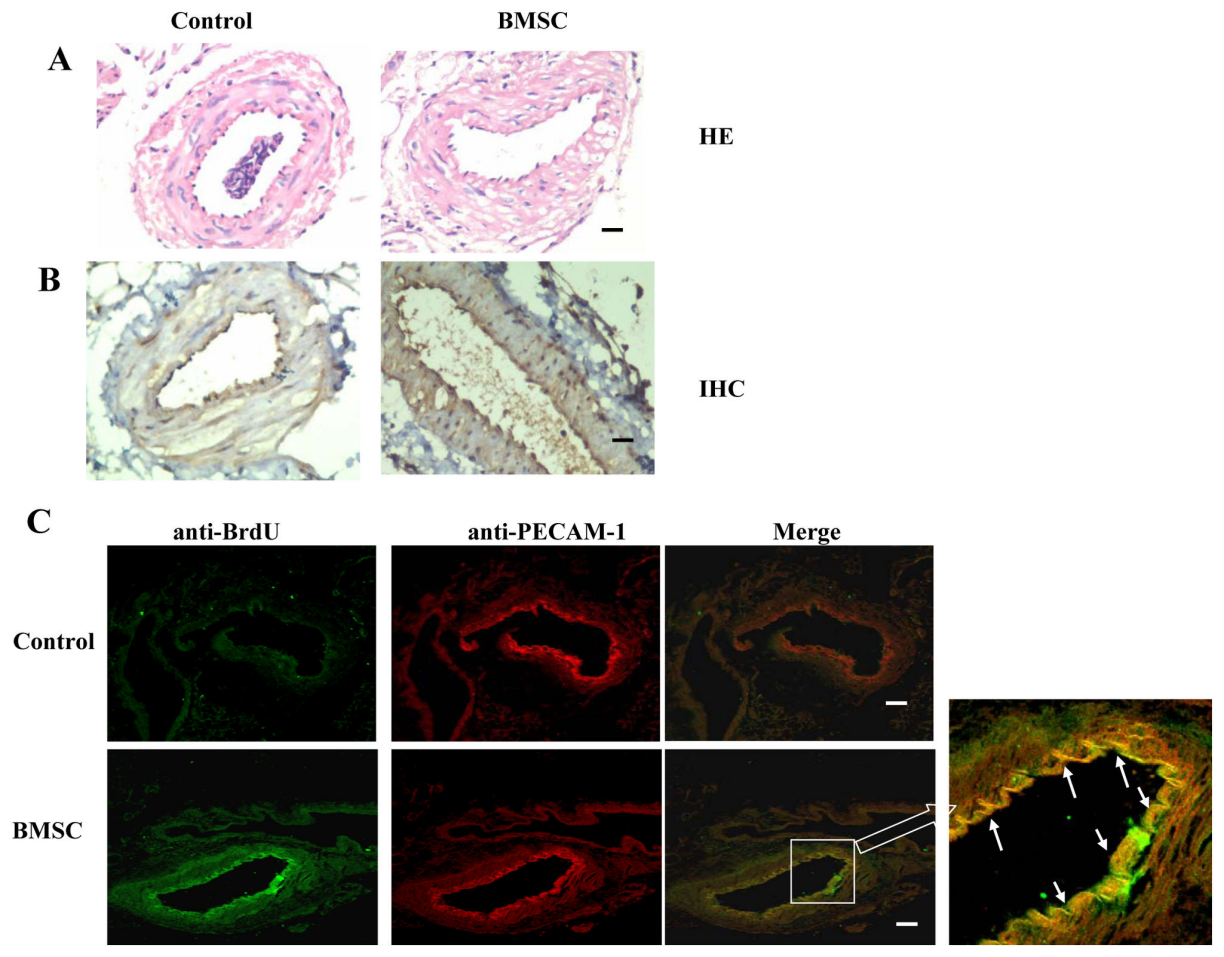
Transplanted BMSCs contribute to neointimal formation and vascular remodelling . BMSCs were labelled with BrdU and injected by intravenous 2 days after carotid artery injury (n=6). After 2 days, the carotid arteries were harvested. (A) Photographs of the carotid injured-artery stained with H&E. Scale bars represent 100μm. (B) The immunochemistry photographs of BMSCs were detected for anti-PECAM-1 between control and transplanted-BMSCs group. Scale bars represent 100μm (C) The immunofluorescence staining for BrdU and PECAM-1 was performed on the consecutive section in neointimal formation under confocal microscopy. These co-stained cells (yellow, white arrows) showed these transplanted BMSCs merged into neointimal formation and expressed the EC marker PECAM-1. Scale bars represent 100μm.

To evaluate the effect of BMSCs on neointimal formation in vivo after carotid arterial injury, BMSCs were labelled with bromodeoxyuridine (BrdU) and were transplanted to these mice with carotid arterial injury and we investigated neointimal formation. To produce high purity transplanted cells from BMSCs, BMSCs were treated with 10 ng/ml VEGF in DMEM for 3 days. We transplanted the BrdU-labeled BMSCs-labelled by intravenous injection. As shown in Figure 5A, The luminal surface of artery showed neo-endothelial cell in the injured-artery endothelium. The anti-PECAM-1 positive cells were detected on the artery intimal surface (Figure 5B), which indicating the BMSCs-transplanted group showed more neo-endothelial cells on the artery intimal surface. 

We detected whether the BrdU–positive cells expressed some markers in endothelium on the injured-artery by immunofluorescence staining under microphotograph. Some double-positive cells were confirmed in neo- endothelium for BrdU and PECAM-1 (Figure 5C). These results revealed that BMSCs significantly contribute to neo-endothelial formation and repair of injured-artery. 

## Discussion

In the present study, we found that BMSCs formed the tube-like structures, induced the mRNA of cell adhesion molecules for ICAM-1 and VCAM-1 and expressed EC-specific markers, such as PECAM-1, vWF and KDR after VEGF induction. In addition, we showed that SDF-1 induced chemotaxis and migration of BMSCs and TNF-α enhanced their adhesion ability to fibronectin like ECs. Then the local transplantation of BMSCs in vascular injury model in mice could promote the vascular remodeling and neointimal formation. The results of this study suggested that BMSCs could play a key role in neointimal formation and arterial remodeling after injury and would be a good candidate for stem cell transplantation. These findings also indicated that BMSCs had a role for therapeutic implications of vascular diseases such as coronary heart disease and atherosclerosis, and were promising source cells for vascular remodeling to injured vessels.

The stem/progenitor cells derived from bone marrow are importance to the vascular development, homeostasis, and remodeling [1,7,26]. These progenitor cells are released into the blood circulation and become an important source of circulating blood progenitor cells[1]. Recently studies have reported that the circulating progenitor cells increase after cardiac surgery, they mobilize and home to injured sites to regenerating tissues and angiogenesis [27,28]. Increasing evidences indicate that bone marrow is the source of these stem/progenitor cells and produces various kinds of stem/ progenitor cells such as BMSCs.

BMSCs have the potential for the differentiation into a variety of specialized cells such as osteocytes, adipocytes, endothelial cells and smooth muscle cells[29,30]. We found that BMSCs after VEGF stimulation could increase the mRNA levels of ICAM-1 and VCAM-1 and express some EC-specific markers such as PECAM-1, vWF and KDR *in vitro*. Our results were consistent with those reported[13,18,31]. These findings indicated that BMSCs had the potential for the differentiation into endothelial-like cells and had the phenotype of ECs. 

Although BMSCs contains multiple subpopulations which limits their usages in therapeutic potential for the transplantation of cardiovascular diseases, transplantation of BMSCs has made successes in recent years. Bone marrow mesenchymal stem cells (BMSCs) transplantation significantly improved left ventricular function [32]. The bone marrow-derived stem cell could promote early re-endothelialization for the denuded vascular injury and participate in vascular repair [33]. We demonstrated that transplanted-BMSCs were observed in the injury vascular artery and BMSCs contributed to neointimal formation on the vascular injury model experiments.

BMSCs contribute to neointimal formation on the vascular injury, which is attributable, in part, to paracrine effects. BMSCs can secrete cell survival factors under favourable conditions. These secreted cytokines promote survival of surrounding cells via paracrine mechanisms[34]. For example, bone marrow stem cells (BMSCs) protect ischemic myocardium through paracrine effects and transplanted mesenchymal stem cells release soluble factors that contribute to cardiac repair and vascular regeneration[35,36]. The secreted factors may exert their therapeutic benefits from preventing apoptosis in cardiomyocytes adjacent to the infarcted area[37]. We suggest that BMSC may release by themselves paracrine factors able to stimulate angiogenesis.

A panel of genetic, antigenic and functional assays is required to provide optimal characterization of BMSCs[38]. At first, we demonstrated that BMSCs *in vitro* had EC characteristics phenotypes and angiogenic properties based on the following observations. BMSCs expressed the mRNA expressions of ICAM-1 and VCAM-1 and were positive for CD31, vWF and KDR under VEGF-treatment, which indicated that BMSCs had the genetic phenotypes of EC. Secondly, BMSCs had the capacity of chemoattractant, migration and adhesion and displayed the ultrastructure of an intact junction structure like EC, which suggested that BMSCs had the angiogenic properties. The last, transplanted-BMSCs appeared in the injury vascular artery and contributed to neointimal formation on the carotid-injured model experiments, which the functional assay was verified. Our results suggested that BMSCs could induce angiogenesis by incorporation into vascular structures and improve function and survival. These findings are consistent with the reported that Wakitani assumed that autologous BMSC transplantation might be widely used [39].

In conclusion, our study showed that transplanted-BMSCs recruited to the arterial lesion after carotid arterial injury and contributed to the vascular remodeling where BMSC participated in neointimal formation in the early development in vivo. In vitro, we have displayed that BMSCs could express some markers and showed the connection and the microscopic structures like ECs. These findings provided direct evidences to support adult stem-cell-based therapeutic approaches for cardiovascular disease and needed to supply with more important details on targeting therapy.
